# Experimental Research and Computational Analysis of Eco- and Biomaterials

**DOI:** 10.3390/ma17174269

**Published:** 2024-08-29

**Authors:** Tomasz Garbowski

**Affiliations:** Department of Biosystems Engineering, Poznan University of Life Sciences, Wojska Polskiego 50, 60-627 Poznan, Poland; tomasz.garbowski@up.poznan.pl

This Special Issue of Materials is dedicated to the exploration and analysis of eco- and biomaterials through experimental research and computational methods. These materials are becoming increasingly significant as construction materials and load-bearing elements across various engineering and medical applications. Biomaterials, derived from biological sources such as wood-based products and corrugated cardboard, as well as synthetic and natural materials, are notable for their ability to interact with organic tissues. Ecomaterials, encompassing construction materials and textiles, also play a crucial role. Both categories include composite materials known for their unique properties that address complex challenges where traditional materials are insufficient. This collection provides a platform for scientists and engineers to share the latest advancements in theoretical, experimental, and computational studies related to eco- and biomaterials. Key topics include mechanical properties and strength estimation, numerical and analytical homogenization techniques, laboratory research methods, and linear and nonlinear analyses of structures made from these materials. The focus extends to laminated, corrugated, and fibrous materials, emphasizing experimental validation and empirical evidence. Among submissions that contribute to the understanding and application of bio-, eco-, and composite materials, one can also find comprehensive studies that highlight the mechanical behavior and practical applications of these innovative materials.

In the collection, Kopras et al. [1] analyze the deflection behavior of steel support plates used in temporary excavations, integrating experimental and computational methodologies to evaluate mechanical performance under real-world conditions. This research bridges the gap between empirical data and theoretical models, involving large-scale field experiments to measure plate deflections and comparing them with numerical predictions using the finite difference method. Combining traditional patch measurements and advanced 3D laser scanning under various backfill loads offers a comprehensive performance assessment. The numerical results align closely with experimental data, validating the computational approach. Notably, a new deflection limit criterion ($w_{gr}=L/130$) for temporary excavation support plates is proposed, enhancing design and safety assessments.

Though the study primarily addresses steel plates, the methodologies and insights are broadly applicable to eco- and biomaterials. The integration of experimental validation with computational analysis serves as a model for sustainable materials in construction. Findings on deflection and load-bearing behavior contribute to the broader understanding needed for developing and applying eco-friendly and biological materials. The empirical data and computational models offer valuable benchmarks for future research, enhancing the reliability and safety of construction practices involving diverse materials.

Ma et al. [2] investigate the seismic performance of stiffened corrugated steel plate shear walls (CSPWs) under various conditions, including atmospheric corrosion. This research provides insights into the mechanical behavior and durability of materials, contributing to the field of eco- and biomaterials. The study introduces three types of CSPWs: unstiffened (USW), cross-stiffened (CSW), and asymmetric diagonal-stiffened (ASW). Using a comprehensive numerical model validated against cyclic test data, the research analyzes the lateral and seismic performance of these walls under monotonic and cyclic loading conditions. The results show that stiffeners significantly enhance the elastic critical buckling load, initial stiffness, ultimate shear resistance, energy dissipation capacity, and ductility of CSPWs. Asymmetric diagonal stiffeners outperform cross stiffeners in improving these properties and reducing out-of-plane deformation and stiffness degradation.

The study also examines the impact of atmospheric corrosion on the seismic performance of CSPWs, finding that stiffeners help mitigate corrosion effects, with asymmetric diagonal stiffeners being more effective than cross stiffeners. A fitted formula for predicting the ultimate shear resistance of corroded CSPWs is provided, offering valuable design guidance for engineering applications. Ma et al.’s work significantly enhances the understanding of how structural modifications and environmental factors influence steel material performance, offering a methodological framework adaptable for eco- and biomaterials. The integration of experimental validation with computational analysis sets a strong precedent for future research in developing sustainable construction materials with improved mechanical properties and durability.

Chybiński and Polus [3] present an experimental study on the load-slip behavior of aluminum–timber composite bolted connections reinforced with toothed plates. This research is pivotal for eco- and biomaterials, offering valuable insights into the mechanical performance and reinforcement techniques of sustainable composite structures. The study evaluates the effectiveness of reinforcing aluminum–timber connections through laboratory push-out tests using laminated veneer lumber (LVL) panels, aluminum alloy I-beams, and various bolts. The primary aim is to determine the shear resistance and stiffness of the connections with and without toothed plate reinforcement. Results indicate that while toothed plates reduce timber destruction in bearing zones, they do not significantly protect against splitting of LVL slabs in connections using grade 8.8 bolts. However, for connections with grade 5.8 bolts of 10 mm diameter, toothed plates substantially increase stiffness, though at the cost of reduced strength due to faster bolt shank shearing.

This research significantly enhances the understanding of composite materials’ behavior under load, particularly those combining metal and timber. The findings provide a nuanced view of how different reinforcement strategies impact composite connections’ performance, directly applicable to the design and development of eco-friendly composite structures. By demonstrating the practical applications and limitations of toothed plate reinforcement, the study contributes to the broader discourse on experimental research and computational analysis of eco- and biomaterials. It underscores the importance of combining experimental validation with advanced modeling techniques to develop innovative, sustainable construction solutions.

Ciesielczyk and Studziński [4] investigate the failure scenarios of various connection types between thin-walled beams and sandwich panels under horizontal loads, simulating bending and lateral–torsional buckling. This research is crucial for understanding the structural behavior and integrity of these connections, which is particularly relevant to this Special Issue. The study examines standard civil engineering connections such as self-drilling fasteners, bolts, blind rivets, and double-sided acrylic tape, both linearly and pointwise. These connections were analyzed under horizontal loads with constant eccentricity to determine their lateral stiffness, initial and secant stiffness, ultimate capacity, and deformation capacity. The research highlights different failure mechanisms: self-drilling fasteners and bolts penetrate all layers, resulting in significant initial stiffness and capacity but also web bow deformation and section rotation under higher loads; blind rivets fail through clamping arm deformation and facing delamination; and double-sided acrylic tapes primarily fail through detachment.

Ciesielczyk et al.’s work provides essential quantitative data on the mechanical response and failure mechanisms of various connection types, offering insights for the design and optimization of sandwich panel connections in eco-friendly and biomaterial structures. This research establishes a comprehensive understanding of how different reinforcement and connection strategies affect the structural integrity and performance of composite materials, laying the groundwork for developing advanced materials and connection methods to enhance the durability and safety of sustainable construction practices.

Sybis and Konował [5] investigate the influence of modified starch admixtures on the rheological properties and compressive strength of cement composites, highlighting the potential of using natural and biodegradable materials to enhance cement performance. The research examines 17 different modified starches, including chemically and physically modified starches and starch hydrolysates, focusing on their effects on the viscosity, tangential stress, yield point, and plastic viscosity of cement slurries. Additionally, the study looks at the flow of fresh cement slurries and the compressive strength of hardened composites.

The results show that modified starches can significantly alter the rheological properties of cement slurries. For example, the retentate LU-1420-0.5%Ac-R increased flow by 82%, and the retentate LU-1412-R improved compressive strength by 25%. However, some starches, like retentate LU-1422-R and retentate OSA-2.5%-R, decreased compressive strength. Sybis et al.’s work demonstrates that natural starch derivatives can effectively modify cement composites, offering potential applications in sustainable construction. The study provides insights into how different starch modifications influence the mechanical behavior and workability of cementitious materials, supporting the development of greener and more efficient building materials. This aligns with the themes of experimental research and computational analysis of eco- and biomaterials, showcasing the practical applications of biodegradable additives in enhancing construction material performance and sustainability.

Qu et al. [6] provide a comprehensive review of stress-fractional plasticity models, which combine fractional calculus with classical plasticity theories to better model the mechanical behavior of materials. This approach is particularly relevant to eco- and biomaterials, offering advanced methodologies for sustainable materials in engineering applications. Fractional plasticity (FP) models are able to capture the non-associated flow behavior of geomaterials like clay, sand, ballast, and rock, which standard plasticity models for metals cannot accurately represent. The review explores the development of FP models; defines the stress length scale (SLS), crucial for fractional differentiation; and discusses two primary branches: past stress and future reference critical states, with a third branch incorporating both for a more holistic approach.

The research highlights the advantages and limitations of FP models and their real-world applications, offering a robust framework for predicting material behavior under various loading conditions and improving simulation accuracy and reliability. Qu et al.’s review contributes significantly to this Special Issue by presenting advanced models that enhance the understanding and performance of sustainable materials. The insights from these models can be applied to develop more resilient and efficient eco- and biomaterials, promoting sustainable practices in construction and engineering. This work underscores the importance of integrating computational approaches with experimental research to innovate and apply eco-friendly materials.

Ksit et al. [7] examine the impact of water vapor and moisture barriers on the energy efficiency and durability of ventilated partitions in buildings, highlighting the importance of moisture analysis in designing sustainable, energy-efficient structures. Excess moisture can degrade building materials, reduce insulation efficiency, and worsen indoor climates, increasing energy demands. The study uses modern digital solutions to model building partitions over an 8-year period under various environmental conditions, focusing on flexible waterproofing materials and their effect on dew point temperature, air temperature, and relative humidity.

The research provides a detailed numerical analysis of different ventilated partition models, assessing the necessity and effectiveness of vapor barriers in preventing moisture damage. By evaluating these barriers over time, the study offers insights into maintaining long-term durability and energy efficiency. Ksit et al.’s work contributes to this collection by demonstrating how advanced computational techniques can enhance sustainable building materials. The findings support the development of resilient construction practices that reduce energy consumption and improve indoor environmental quality, aligning with sustainable architecture goals.

The article by Malewski et al. [8] presents developments in the constitutive material model for architectural soda lime silicate (SLS) glass, updating key modeling parameters. This research is crucial for eco- and biomaterials, providing insights into precise material parameters for accurate numerical modeling of glass in sustainable architectural design. By conducting experimental investigations on the coefficient of thermal expansion (CTE), glass transition temperature, and Young’s modulus, the study addresses gaps in contemporary data on SLS glass. These parameters are critical for predicting glass behavior under various conditions, including mechanical and thermal performance.

Malewski et al.’s work significantly enhances the understanding of glass behavior under different environmental conditions. The updated material parameters improve simulations, design, and prototyping of complex glass structures, leading to more efficient production and supporting sustainable building practices. Integrating experimental data with computational analysis, the study bridges the gap between theoretical models and practical applications, ensuring materials used in sustainable construction are reliable and efficient. This research underscores the importance of continuously updating material databases to reflect current technologies and practices, which is essential for the ongoing development of sustainable architecture.

The article by Mrówczyński et al. [9] investigates the compressive strength capacity of open-top corrugated board cartons used for transporting fruits and vegetables. This study is highly relevant to eco- and biomaterials, focusing on optimizing biodegradable and sustainable packaging. Using sensitivity analysis, the research identifies critical geometric parameters affecting load capacity. The authors developed a finite element model to simulate the box compression test (BCT) and analyzed seventeen design parameters. They found that modifications in the length of non-folded sidewall parts and box height significantly influence compressive strength, while dimensions and positions of ventilation holes had negligible effects.

By identifying key parameters affecting structural integrity, the study provides valuable insights for designing eco-friendly packaging solutions. The findings help manufacturers create more efficient and durable corrugated board cartons by focusing on influential geometric factors, thus reducing material usage. Mrówczyński et al.’s work demonstrates how advanced computational techniques and sensitivity analyses optimize biomaterial design. This approach enhances the mechanical performance of materials and supports environmentally friendly packaging solutions, reducing the ecological footprint of the packaging industry. Integrating experimental validation with computational modeling sets a robust precedent for future research in sustainable materials.

The article by Fehér et al. [10] explores the compression strength estimation of corrugated board boxes, focusing on reducing sidewall surface cutouts. This research is relevant to the Special Issue as it optimizes biodegradable packaging materials to enhance mechanical properties while minimizing material usage and waste. The study investigates various cutout configurations on B-flute corrugated cardboard boxes, commonly used in supply chains, and compares experimental observations with the McKee formula for compression strength estimation.

Fehér et al. found that compression strength decreases linearly with increasing cutout size, showing significant discrepancies from the McKee formula, which doesn’t account for sidewall cutouts. To address this, the authors propose a modified estimation approach incorporating empirical test data for more accurate predictions. This research demonstrates how experimental and numerical methods can optimize sustainable packaging design, providing valuable insights for packaging engineers to balance material reduction with mechanical performance. The integration of empirical data with computational analysis exemplifies the interdisciplinary approach necessary for advancing sustainable materials in practical applications.

Mrówczyński and Garbowski [11] explore the influence of imperfections on the effective stiffness of multilayer corrugated board, which is crucial for understanding the structural integrity of eco-friendly packaging materials like corrugated cardboard. Widely used for its recyclability, biodegradability, and durability, corrugated cardboard is an increasingly popular choice as companies move away from plastic to improve their environmental impact. The study focuses on how geometric imperfections from the manufacturing process affect the mechanical properties of cardboard.

The authors present a numerical homogenization procedure using the finite element method (FE) to include geometric imperfections in calculating the board’s effective stiffness. A 3D model of a representative volumetric element (RVE) is built, incorporating various buckling and distorted shapes from prior analysis. This approach allows for a quick and scalable method to account for imperfections and their impact on material stiffness. Mrówczyński et al.’s work significantly contributes to this collection by providing a framework to understand how imperfections affect the mechanical behavior of sustainable packaging materials. The findings help optimize the design and manufacturing processes of corrugated cardboard, ensuring its integrity and performance despite manufacturing-induced imperfections, supporting the development of resilient and efficient eco-friendly packaging solutions.

Fehér et al. [12] investigate the compressive strength of corrugated paperboard packages with varying cutout rates, which is integral to understanding eco- and biomaterials. The study uses the finite element method (FEM) to model and predict the compression force of corrugated cardboard boxes with different sidewall cutout configurations. Boxes tested had widths and heights of 300 mm, lengths from 200 mm to 600 mm, and cutout rates of 0%, 4%, 16%, 36%, and 64%. The FEM model incorporated a homogenized linear elastic orthotropic material with Hill plasticity.

Results from numerical simulations and experimental box compression tests (BCT) showed that the FEM model accurately predicts the compression strength across various cutout configurations. However, model accuracy slightly decreased with higher cutout rates, highlighting the challenge of maintaining structural integrity. Fehér et al.’s work contributes significantly to sustainable packaging by providing a reliable numerical model for predicting the performance of corrugated cardboard boxes with different cutout designs. This research helps optimize eco-friendly packaging design and manufacturing, ensuring strength and durability while reducing material usage and waste.

Bartkowiak and Słowik [13] focus on developing a predictive model for the stiffness modulus $\left|E^{*}\right|$ of high-modulus asphalt concrete (HMAC) using the four-point bending beam test (4PBB). This study is relevant to eco- and biomaterials, offering insights into optimizing sustainable infrastructure materials through advanced modeling techniques. Building on existing models like the Witczak model, the authors aimed to enhance predictions for HMAC’s stiffness modulus. This involved extensive laboratory testing of asphalt mixtures to gather data on stiffness modulus and phase angles under varying temperatures and loading frequencies, including tests on neat and modified bituminous binders using a dynamic shear rheometer (DSR).

The research presents a new model, Model A, which modifies the Witczak model to improve accuracy for the 4PBB method, considering air void content, effective binder content, and aggregate gradation. Results show the model predicts stiffness modulus with high accuracy, as evidenced by low errors. This work contributes to the Special Issue by demonstrating refined computational models that enhance understanding and performance of sustainable construction materials. The findings support developing more resilient and efficient infrastructure using eco-friendly materials like HMAC, aiding engineers in designing and optimizing asphalt mixtures for better durability and performance, thus contributing to sustainable, long-lasting infrastructure solutions.
